# Motivation to smoking cessation and working conditions among healthcare workers

**DOI:** 10.1192/j.eurpsy.2026.10817

**Published:** 2026-07-31

**Authors:** I. Sellami, A. Abbes, A. Feki, M. L. Masmoudi, M. Hajjaji, K. Jmal Hammami

**Affiliations:** 1 occupational medicine; 2 Rheumatoloy, Hedi Chaker Hospital, sfax, Tunisia

## Abstract

**Introduction:**

In the professional environment, a smoke-free workplace is essential to ensure the health of employees and those around them. Understanding the link between the motivation to quit smoking and working conditions is a key factor in shaping effective anti-smoking measures for healthcare workers.

**Objectives:**

This study aimed to investigate the relationship between motivation to smoking cessation and working conditions among healthcare workers.

**Methods:**

The study was carried out among a sample of healthcare workers. Data collection was carried out using a self-completed questionnaire. We collected socio-professional data. We used the single-item measure of job satisfaction, the work ability score and the borg scale to evaluate physical strain. Motivation to smoking cessation was rated on a 0–10 scale, with 0 representing low and 10 representing high motivation.

**Results:**

Our study population included 57 healthcare workers, 52.6% of whom were women. The mean age of participants was 36.1 ± 10.2 years. Their mean of job tenure was 15 ± 11.5 years, and the mean of job satisfaction score was 3.4 ± 1.2. The mean of work ability was 7.9 ± 2 and mean of physical strain was 9.2 ± 1.1. We found that 15.8% of participants were active smokers and 14% were former smokers. The mean of motivation to smoking cessation was 7.4 ± 4. High motivation to smoking cessation was associated to higher job satisfaction (p=0.03, r= 0.71).

**Conclusions:**

The association between smoking cessation and job satisfaction highlights the need to undertake preventive actions that mitigate occupational burdens, and enhance job satisfaction in conjunction with smoking cessation interventions for healthcare workers.

**Disclosure of Interest:**

None Declared

